# The Hospital Nursing Supplement

**Published:** 1896-03-07

**Authors:** 


### "The Hospital" Nursing Mirror.

BEING THE EXTRA NURSING SUPPLEMENT OF "THE HOSPITAL" NEWSPAPER.

[Contributions for this Supplement shou.d be addressed to the Editor, THE HOSPITAL, 428, Strand, London, W.C., and should have the word "Nursing" plainly written in left-hand top corner of the envelope.]

### News from the Nursing World.

### ROYAL VISITS.

ON Friday, February 28th, the Duke and Duchess of York visited the Victoria Hospital for Children at Chelsea, and the Princess May presented each child with a toy. The wards looked very bright and cheery, and pretty little occupants were in possession of the Royal cots, the "Prince Eddy" and the "Prince Edward of York." The Duke and Duchess, who expressed themselves pleased with their visit, afterwards had tea with Miss Hamilton, the matron. H.R.H. the Prince of Wales paid quite a lengthy visit to the Alexandia Hospital for Children at Brighton on Sunday last, remaining some time in the wards.

### LONDON HOMŒOPATHIC HOSPITAL.

THE Duchess of Teck has consented that the east wing of the new building of the London Homœopathic Hospital, opened by Her Royal Highness in July last, shall be named "The Princess Mary Wing." The west wing will be named "The Durning Wing," by permission of a liberal donor.

### ROYAL FREE HOSPITAL MEDICAL SCHOOL.

MRS. PERCY FLEMMING, M.D., and Miss Aldrich-Blake, M.S., have been appointed medical and surgical registrars respectively at the Royal Free Hospital. It is the first time these appointments have been held by women. Miss Aldrich-Blake is also the first woman to obtain the much-prized diploma of M.S.Lond. The report of the medical school for 1895 is in every way a highly satisfactory one. Twenty-five students have qualified as medical practitioners during the year, and several former students have distinguished themselves in various examinations, five having obtained the M.D., seven the M.B., and five the B.S. degree of the London University.

### EAST LONDON HOSPITAL FOR CHILDREN.

THE nurses at the children's hospital at Shadwell, if their outside surroundings are not attractive, are well cared for in the matter of internal accommodation, and the various arrangements for the comfort and well-being of the nursing staff generally were pointed out with pride by the bright and courteous nurse who took certain inquiring visitors under her kindly guidance on the reopening day last week. The sleeping quarters are divided from the hospital proper by a passage and private door, and consist of very pleasant, light, and airy cubicles, which are, by the way, quite large enough to admit of the partitions being carried up to the ceiling in each case, as has already been done for the sisters' rooms, an improvement which, no doubt, will be effected by-and-bye. For cubicles, however, these are most comfortable, and are very nicely furnished. The bath-room and lavatory accommodation, too, is good. The paying probationers have similar quarters on the top floor of the hospital. The nurses' sitting-room is a cosy little apartment, with plenty of easy chairs, and the arrangement by which the large and pleasant board-room is utilised as a dining-room for the nursing staff is excellent, and one which might be well followed in other hospitals where room is limited and where such a plan would result in greatly-increased comfort for the nurses. One side of this room is devoted to the nurses' library, being fitted with handsome bookshelves, for which, no doubt, additions would be gladly received. The sisters, whose bed-rooms were formerly partitioned off the wards, have now rooms allotted to them at the end of the nurses' corridor, being also provided each with a tiny though cosy sitting-room leading from their ward. Miss Row and her staff must have felt justly proud of their charming flower-decked wards the other day, which certainly received a full meed of appreciation from many admiring visitors.

### GUY'S HOSPITAL LADIES' ASSOCIATION.

A USEFUL organisation exists in connection with Guy's Hospital bearing the above title. It has for its objects (1) making and providing of clothes to be lent or given to destitute patients on convalescence or discharge; (2) friendly visiting in the wards by the members, subject to certain rules; and (3) the adoption of any work for the good of the hospital which may commend itself to a general meeting of the association. Mrs. Randall Davidson is the president, Mrs. Pye Smith the treasurer, and Mrs. Lauriston Shaw (10, St. Thomas's Street, S.E.) the secretary. Members may be honorary or working, the former paying an annual minimum subscription of half-a-guinea, the latter of half-a-crown, and these last are expected to take an active share in the work of the association. Working parties are held at the houses of members, the distribution of the garments there made is placed in the hands of the matron and sisters. The secretary (to whom communications should be made in the first instance by ladies wishing to join) will also welcome gifts of old clothes, especially for men and boys, or flowers and fruit for the patients.

### A PENSION FUND NURSE.

THE recently-issued annual report of the Liscard and New Brighton District Nurse Fund, which is by the way a particularly satisfactory one, contains a special paragraph to the following effect: "It may be interesting to subscribers to know that Nurse Coffey, being an insurer in the Royal National Pension Fund for Nurses, originated in connection with the Queen's Jubilee" (there is some slight confusion of ideas here apparently with the Q.V.J.I.N.), "was among the nurses who were honoured during the summer by an invitation from the Princess of Wales. . . . They were most kindly received at Marlborough House, were addressed by the Prince of Wales, and received certificates and badges from the Princess." Truly the pleasure given to many a hard-working nurse by that same gracious reception of "Our Princess" would be difficult to over-estimate, and the fact that "nurse's invitation" finds a place in the report of the year's proceedings shows, too, how widespread is the interest taken in nurses and their general welfare.

### A GERMAN UNIVERSITY FOR WOMEN.

A good deal of interest is being taken in Germany in the questions raised in England with regard to the admission of women to University degrees. Dr. Dernburg, a well-known professor of law at Berlin, holds that women who "have the requisite capacity for academic study have also the right to it, but that it is quite another question whether it would be wise to admit both sexes indiscriminately to the German Universities." He rather would suggest that one of the Universities, "Giessen, for instance, beautifully situated in the middle of the Empire," should be especially devoted to women.

### QUEEN'S NURSES, INVERNESS BRANCH.

An amateur musical and dramatic entertainment, got up the other day at Inverness, in aid of the local branch of the Queen's Jubilee Institute, has resulted in a sum of £25 being placed to the credit of the institution. Being a drawing-room entertainment expenses were but small, and almost the whole of the proceeds were therefore devoted to the charity.

### MISS BLENNERHASSETT IN SOUTH AFRICA.

The home for consumptive invalids and convalescents started at the Poplars, Middelburg, Cape Colony, by those two energetic nurses, Miss Blennerhassett and Miss Sleeman, is now being recognised as a really public boon. Middelburg is 4,000 feet above the sea, and is recommended strongly by the medical profession as a health resort. The names of these two English nurses sufficiently guarantee that every comfort is provided for the invalids under their care, and there is an English doctor and an English clergyman in Middelburg, which boasts a railway station, a public library, billiard-rooms, and tennis courts.

### FIRE AT A LIVERPOOL HOSPITAL.

An alarming fire broke out at the Liverpool Eye and Ear Infirmary last week, and resulted in a good deal of damage to the institution. The flames were first seen issuing from the dining-room on the first floor, and most of the inmates had to be rescued by means of a ladder, most valuable and prompt help being rendered by the neighbours before the appearance of the fire brigade. As there were in the hospital at the time some twenty-two patients, the matron, eight nurses, five servants, and a porter, the task of removal must have been no light one. Only two cases of injury are reported, and one at least of these would not have occurred but for a very lamentable want of self-control on the part of one of the nurses, who threw herself out of an upper window, breaking her leg and injuring her spine. The other victim was a patient, who burnt her foot while endeavouring to make her way down the stairs. It is fortunately seldom that nurses, trained as they are to calmness and self-effacement, lose their heads in times of danger, for on their coolness rests to a large degree the safety of the sick in their care by the steady maintenance of discipline in an emergency, and a nurse's first thought should be, and usually is, the care of her patients. Uncontrolled terror on her own account may at such times have the most disastrous effects, and is most unfortunate in the case of anyone in a position of responsibility.

### THE TRAINED NURSES' CLUB.

The next lecture at the Trained Nurses' Club, 12, Buckingham Street, Strand, will be given on Friday, March 13th, at a quarter to eight, by Mrs. William Archer, the subject being "Nerves and a Little Common Sense." The lecture will be especially interesting to masseuses, and members of the Society of Trained Masseuses are invited to be present.

### QUEEN'S JUBILEE NURSES AND THE PENSION FUND.

Great misapprehension has been caused by the announcement that "Mr. Henry Tate has given £5,000 to Queen Victoria's Jubilee Institute for Nurses, which provides the poor with nurses in their own homes and also a pension fund for nurses." As a matter of fact, there is not, and never has been, any question of a special pension fund in connection with the Queen's Nurses. They are, however, encouraged by the institute authorities to join the Royal National Pension Fund for Nurses, which fulfils every requirement in the matter of nurses' pensions; and, though the Queen's Jubilee Institute is not as yet affiliated with the Royal National Pension Fund, that is a step which it hopes to accomplish in the future. Mr. Tate gave his £5,000 for the benefit of the nurses, and it is most likely, we hear, that it will be used in aiding nurses to avail themselves of the Royal National Pension Fund, or for some such benevolent purpose.

### UNIVERSITY DEGREES FOR WOMEN.

On Tuesday, at the largest Congregation of the University of Oxford that has ever met, the following resolution was proposed by the Rev. T. H. Grose, Fellow of Queen's College, President of the Association for the Education of Women: "That it is desirable, subject to certain conditions, to admit to the degree of B.A. women who have kept residence at Oxford for twelve terms, in a place of residence approved by the University, and who have passed (under the same regulations as apply to undergraduates) all the examinations required for the degree of B.A." After some discussion a division was taken, and the resolution was finally thrown out by a majority of 75. The consideration of the further question of granting diplomas and certificates to women was then postponed by the Vice-Chancellor to next Tuesday. At Cambridge the proposal to appoint a syndicate to consider the question of degrees for women will be submitted to the Senate on March 12th.

### SHORT ITEMS.

It is stated that Miss Clara Barton has succeeded in obtaining an interview with the Turkish Minister of the Interior, and, having received permission, has journeyed on into Armenia with her party to carry out her mission of relief to the destitute inhabitants.—Miss Morgan, sister of the Broderip Ward at Middlesex Hospital, has been offered and has accepted the appointment of matron of the new convalescent home in connection with the hospital at Clacton-on-Sea, which is to be opened this summer.—Miss Margaret David on, who holds the certificate of the Mater Misericordiæ Hospital, Dublin, has just been unanimously elected head-nurse of the Ballieborough Workhouse Infirmary.

### Lectures on Nursing.

By a Superintendent of Nurses.

### XII.—OBSERVATION.

It is essential that a nurse should cultivate the habit of observation, as nothing connected with a patient or his surroundings is too trivial for notice. A very slight change may indicate a serious condition of things, and it is of the utmost importance that the doctor should be told everything that can help him to diagnose a case. The nurse should carefully notice the general appearance of a patient, whether flushed, anæmic, or blanched as if he had lost a large quantity of blood; whether feverish or faint; whether tremulous, as if suffering from some nervous complaint; whether there is a look of horror, dread, and suspicion, the peering round the door, &c., as in delirium tremens; whether the expression on the face is calm or excited: whether there is the drawn face, the limp condition of the limbs on one side of the body, as in hemiplegia; whether there is the hollow cough, the hectic flush of the phthisical patient; the convulsive seizures and struggles, the upturned eyes, the foaming at the mouth, the grinding of the teeth, the biting of the tongue, the livid appearance often accompanied by the peculiar shriek of epilepsy; the stertorous breathing, the florid face and unconsciousness of the apoplectic; peculiar involuntary muscular movement of chorea; the puffy face and pallor of chronic Bright's disease; whether there is any scarlet rash, as in scarlet fever; a red-mottled crescent-shaped rash as in measles, or a rash composed of small vesicles, some of them perhaps having crusts upon them, as in chicken pox. His position in bed (decubitus), whether he lies in a flat immobile posture, with his face to the ceiling as in typhoid fever; whether he lies on the affected side so as to leave the other free from embarrassment, as in pleurisy; whether he sits bolt upright in bed, gasping for breath, probably with cyanosis of face, and general look of anxiety, denoting, perhaps, advanced stage of cardiac trouble; or if with flushed perspiring face and rapid shallow breathing of pneumonia; whether he lies on his back with a look of suffering on his face, with knees drawn up, indicative of peritonitis. His skin, whether cold, clammy, hot, pungently hot, dry, or moist; any peculiar odour, the fœtor of the gangrenous lung or of malignant disease—the sweet, hay-like smell of diabetes. The pulse, whether quick and strong as in fever, or quick, full hard, as in inflammation; whether rapid, small, or "thready," as in a low, debilitated condition; whether jerky, giving a quick bound and then suddenly ceasing, as in some valvular diseases of the heart; whether an intermittent pulse, in which a pulsation is omitted every now and then, the number of beats differing one moment from that in another, as in some diseases of the heart; whether it is hard and difficult to compress, or wiry, *i.e.*, hard, yet small.

In taking the pulse the nurse should see that the patient is as restful as possible, and has not been exerting himself for some time previous; she should distract his thoughts, if possible, so that he does not think of his pulse. The arm should be resting on the bed, and she should apply the tip of the first finger of her right hand to the front of the forearm, one inch above the wrist, and about half an inch from the outer edge. There should be no pressure on the artery in any part of its course between the wrist and the heart. The watch used by the nurse should have second hands, and she should count the number of beats in thirty seconds and then multiply by two. The pulse of an adult beats 70 to 80 a minute on an average, that of an infant 100 to 120, that of a child 80 to 100.

The temperature: This is of the utmost importance, being a most valuable index to the state of health, more especially in specific fevers, ague, midwifery practice, and after operations.

Every nurse is familiar with the clinical thermometer generally used in England, which is graduated according to Fahrenheit's scale, that is, 32 deg. freezing and 212 deg. boiling-point, but the index only ranges between 96 deg. and 110 deg. Each of the smaller divisions of the scale $= \frac{2}{10}$ $= {\cdot}2$ of a degree, two divisions therefore $= {\cdot}4$, three divisions $= {\cdot}6$, four divisions $= {\cdot}8$. The bulb of the thermometer should be as thick only as the diameter of the stem, and the index a thread of mercury detached from the bulb by means of an air bubble. Before taking the temperature the index should be shaken down to 96 deg. This is especially necessary where children are concerned, becaause their temperatures are so frequently subnormal. Care must, however, be taken not to drive the index down too violently for fear of sending it into the bulb. Five minutes should always be allowed in registering a temperature, and it should always be taken in the same place in the same patient, as it varies according to where it is taken, being higher in the internal than in the external parts of the body.

The axilla, as a rule, is the best place in which to take the temperature, but if it is moist with perspiration the part shoud be carefully wiped dry before putting the thermometer under the arm. It is best for the patient to lie partly on his side and partly on his back during the time of observation. If the temperature is taken in the mouth, the bulb should be put under the tongue, the lips should be tightly closed, so as to exclude the cold air, the teeth being lightly closed, so as not to bite the bulb off; the stem should be held by the hand of the nurse, or of the patient himself if well enough. When finished with the thermometer should be well washed in carbolic. With young children it is rarely safe to put it into the mouth, so the temperature is usually taken in the rectum. The child should be placed on its side and prevented from moving, and the bulb introduced for about an inch, the stem being held to steady it—or the temperature may be taken in the groin, the child lying on its back, and the thigh being flexed on the abdomen.

The normal temperature is 98·4 deg., but higher usually with children; and if it rises above 99·5 deg., or falls below 97 5, the presence of disease is indicated. It rises from morning till midnight, and is lowest about six a.m. When it is above 100 deg. it is spoken of as pyrexia, and when it reaches above 105 it is called hyper-pyrexia. This usually occurs only in rheumatic fever, or in spinal injuries, when it may go up to 108 deg., or even higher, but death almost always follows. When above normal it should always be taken four times a day, and charted, or entered at once in a book, as a nurse should not trust to her memory. Respiration: This should be noticed by the nurse when the patient is in a restful condition, and when he is unconscious of her observation if possible, as the knowledge of her watching him for this purpose may alter the rate. She can count the inspirations whilst her attention seems to be on the pulse. The normal rate varies somewhat, but is usually about seventeen respirations per minutes. If difficult to count without doing so, she can place her hand on the chest, and on the abdomen of children. In some cases the nurse may notice a peculiarity in breathing; the patient may inspire at first lightly, then deeper and deeper until he reaches a maximum, then the inspirations will be feebler and shorter until he seems to stop breathing altogether, and may continue in this condition for so long as to alarm those about him; then suddenly a low inspiration will be noticed, followed by fuller ones. This occurs in some brain diseases, and is called the "Cheyne Stokes" breathing. It is a very bad symptom.

The tongue: Notice whether it is moist or dry, whether swollen and flabby, indented by the teeth, the colour like strawberries, as in scarlet fever, whether glazed as in diabetes, whether when put out it protrudes on one side of the mouth, whether dark-coloured and cracked, as in some fevers, or with small ulcers, whether there is a thick black fur with sordes on the teeth, whether a white fur as in inflammations.

The mental condition: If the nurse notices any sign of mental aberration, she should most carefully remove anything that may suggest or be used for self-destruction; all instruments, even needles, string, &c. I once knew a girl who, having gone through a great deal of suffering, attempted suicide by taking some of the bandage off her leg one night, and fastening it to the top of her bedstead, wound it round her neck with the intent to destroy herself. Fortunately the night nurse found this out in time to save her, but not before her face was livid.

### Homeward Bound.

(A Letter from Our Indian Correspondent.)

We have had an interesting, though a very long, journey home, having left Umballa in the middle of December, and only arriving in England at the beginning of last week. I visited Delhi and Agra on my way from Umballa, being joined at the latter place by a friend, with whom I travelled down to Bombay. There we did a good deal of sight-seeing, and then, when we finally embarked on board the "Victoria" two days later, we sailed for the Cape, instead of home, being commanded by the Admiralty to await orders at Cape Town. Visions of possible volunteering in Ashantee flashed before us, for we thought it might be that we were to take troops to Cape Coast Castle, and so might see a little active service. But when we reached Cape Town no orders had arrived. Troops were concentrating there, and the greatest excitement prevailed about affairs in the Transvaal. While waiting at the Cape we visited several hospitals, and especially admired the New Somerset, which seemed to me, as far as a casual visitor could judge, to be quite perfect. Shops and streets looked very English, and we enjoyed the theatre, which was in possession of a London company. The country and scenery round Cape Town are delightful. There is abundance of lovely flowers and wonderfully cheap fruit; peaches, pears, and grapes were in season, the former costing only fourpence a dozen.

On the sixth day we were ordered back to Durban to take up Dr. Jameson's party, going this time up the other side of Madagascar. We were much excited, for at the Cape Dr. Jameson was the popular topic, his photograph was in every window, and his portraits painted on ostrich eggs were selling at 1s. 6d. each. We learnt a good deal of the feeling about him in Cape society. Everyone who has known him speaks well of him as a good man, and most highly of his professional abilities.

Between Cape Town and Durban we suffered a good deal from heat and rough weather. There is a bar across the harbour at Durban so that big ships cannot enter the dock, and the only way of getting ashore is to be slung off in baskets, three or four at a time, on to a tug, luggage being embarked and disembarked in the same fashion. The prisoners came alongside in a tug early in the morning with a guard of officers of the 7th Hussars. Many of them were very sea-sick, but notwithstanding that disadvantage they made a very striking picture in their Buffalo Bill sort of get-up. Dr. Jameson and Colonel Grey, who were in plain clothes, looked very dejected, and so did Sir John Willoughby. Captain Gordon Wilson and Lady Sarah Wilson, who had joined us at Cape Town, left the boat at Durban, going in the baskets, but those who left the ship later were obliged to descend rope ladders into small boats to be conveyed to the tug, the sea being too rough to allow it to come alongside.

We saw a good deal of Dr. Jameson's party. They did not dine in our saloon, but many of them sat with us in the music saloon and used our deck. Some of them we grew to know very well, but Dr. Jameson himself, and Sir John Willoughby and Colonel White, kept very much to themselves, and did not cultivate ladies' society. I liked what I saw of Dr. Jameson very much.

We stopped at Zanzibar for three days, which we found very interesting. Here the slave trade still exists; the slaves are the beasts of burden, the Arabs the masters. We visited one of the Sultan's palaces (he has three) and saw the Sultan parade his troops at half-past six p.m., at which hour every day he shows himself to his subjects. Every morning he fixes the time of day, and the clock is put accordingly. When we were there one side of the clock was half-past six (our time) and the other half-past eleven (his time). The streets in Zanzibar are very narrow passages, only one being fairly wide, and no one keeps carriages but the Sultan; he lent us one occasionally. The island is very tropical in character, covered with plantains, dates, palms, and ferns. Zanzibar has a good cathedral. We invested in many curios, for there was a great sale of ebony and ivory. Our next stop was Suez; then on to Port Said and Malta, and we finally reached Plymouth on February 24th.

### Royal National Pension Fund for Nurses.

THE NURSES' GIFT TO H.R.H. THE PRINCESS MAUD OF WALES.

Nurses who may not have seen our announcement under this heading last week will be glad to be reminded that the beautiful tea table which is to be their wedding gift to the Princess Maud will be on view at 5, Bond Street, by permission of Mr. Albert Barker, the maker, until March 9th. We have been very pleased by several cordial expressions of approval from nurses who have inspected the table and its dainty accessories. The following has just reached us: "The Leeds district nurses beg to say how very much pleased they are to learn that the Princess Maud's wedding gift has been so successfully executed, and to express their satisfaction and thanks to those who carried out the work so well."

### Royal British Nurses' Association.

We are requested to insert the following letter from Nurse Groom, addressed to the secretary of the Royal British Nurses' Association, as it expresses the feeling of various members of the association that a special fund should be started to defray the recent heavy legal expenses in which the association has been involved: "I enclose the ten shillings according to promise on behalf of Nurse Collingwood and myself, and I think if you acknowledged it in The Hospital it might cause others to subscribe. It was through reading Sir Dyce Duckworth's letter in The Hospital that we thought of helping to pay the expenses. I hope the whole of the £150 will be subscribed by the nurse members, who will thus show their loyalty to our gracious president, H.R.H. Princess Christian, and to the association."

### Appointments.

National Hospital for Consumption for Ireland, County Wicklow.—Miss Jessie Gertrude Powell has been appointed lady superintendent of this hospital. Miss Powell was trained at Sir Patrick Dun's Hospital, Dublin.

### Superintendents of Training Schools' Association.

By an American Correspondent.

MEETING IN PHILADELPHIA, 1896.

The third annual convention of the American Society of Superintendents of Training Schools for Nurses was held in Philadelphia February 11th, 12th, 13th, and 14th, 1896. The Hotel Colonnade was selected as the place of meeting. The council met Tuesday evening, and Wednesday and Thursday mornings were devoted to the general sessions. About fifty superintendents answered to the roll-call, and the programme, which had been carefully prepared, was carried out in full, not one failing in the duty assigned her. Mrs. Hunter Robb and Miss Irene Sutliffe sent messages of regret for enforced absence, and the secretary was instructed to make suitable replies.

The society was welcomed to Philadelphia by the president (Miss M. E. P. Davis). After considering the absolute necessity of raising the standard of training, Miss Davis said: Our path lies straight before us—to point out the defects of our present method, or lack of method; to arrange a system that can be applied, with but slight variation, to the individual needs of both large and small schools; to agitate the matter; to get it to the ear of the School Boards; to discuss the question with the individual members of our own board with whom our opinions and ideas have weight; to "talk shop," if necessary, to members of other boards whom we may meet either socially or officially; to keep before the minds of the people who are engaged in philanthropic and educational work; to be enthusiastic about it; to be unflagging in our efforts to inspire confidence that what we want is right and just; finally, to do all this in such a way that everyone who hears our arguments will be convinced that we are doing this not for our own aggrandisement or pleasure, but because it is our honest conviction that if we can secure the co-operation of our boards and establish a universal system of training the greatest obstacle to the elevation and advancement of the profession will be removed.

Twenty-seven active and two associate members were elected, and it was voted that there should be a third class known as visiting members.

Miss L. L. Drown reported for the committee on "Eligibility for Membership." This report was so brief that we give it in full: "This committee recommends that for full membership a candidate must be a graduate of a school connected with a general hospital of not less than fifty beds, giving not less than two full years of training in the hospital; also that she must be the superintendent of a training school complying with the same conditions, and that she shall be acceptable to the society. The committee recommends that there shall be two classes of associate members, the second as voted to be known as visiting members. First, assistant superintendents as provided in article five of the constitution, it being understood that the superintendent of the school is eligible for full membership. Second, graduates of a school where the superintendent is eligible for full membership who may be in charge of small general hospitals of not less than twenty-five beds, or special hospitals, all such schools giving not less than two full years of instruction, such members to be acceptable to the society; such members not to be entitled to vote or hold office. The initiation and annual fees for visiting members to be the same as for associate members."

Miss Snively, for the committee on a uniform curriculum, made a model report, far too long and carefully prepared to be embodied in a mere synopsis of the work of the convention. Special stress was laid on the need of spending two years in a general hospital of not less than 150 beds, or three in one of 75 beds, and on uniform entrance examinations. The course as mapped out by Miss Snively has been used by her with satisfaction during the past year. It was proposed that a copy of this proposed curriculum should be sent to all superintendents of training schools with the request that they should try it for a year and report on it at the next annual meeting.

Several papers were presented to the society, one on training school registries by Miss Darche. The Bellevue School and the Illinois School Registries were cited as good examples. Both are controlled as a part of the school management. The graduates' co-operative registry is still in the future, but I hope in the near future. The first step towards such a registry should be a graduates' club. It is most important that the physicians or other patrons of the registry should understand from the first that good nurses can always be obtained by writing, telephoning, or calling at the registry. It is not necessary that applicants for nurses should see or talk with the nurse, providing the agent can guarantee a good one. They should never be obliged to look up the nurse herself at her lodgings. Many nurses object to having a price fixed for their services. They say they should do as the doctors do—charge according to the fortune or income of the patient's family, or the difficulty of the case. This is a fallacy. There should be a definite fixed charge known from the start. Probably the best schedule of rates is that fixed on the graded basis. We who, as superintendents, have gained some experience in management and organisation, owe it to our graduate nurses in the work of nursing, so far as in us lies, and with all the influence our position gives, to teach them, for their own safety and protection, to co-operate and organise societies for mutual protection and advancement.

One of the most valuable papers, and one involving a great amount of work, was prepared by Miss Nutting, "A Statistical Report of Working Hours in Training Schools." The statistics were gleaned from 111 training schools in England, Canada, and America. The working day from the hour of rising varies from 15½ to 17 hours, the average day being 16½ hours, the rising hour six a.m., the retiring hour half-past ten, allowing 7½ hours for sleep. The hours actually on duty in the wards vary from 8 to 15 hours. In the greater number of hospitals the nurses are on duty 10½ hours daily. The pupil in a training school may work harder to receive her training than a labouring man to support his wife and family, for here we find in one of the most difficult works a woman can undertake—and her only method of receiving a certain kind of education—it is not a 60 hour week, but one varying from that number to 105 hours. In those schools where the very shortest hours are adopted nurses are working nine hours a day in work which taxes the physical strength even of the strong in no small degree, of the moderately strong to the utmost. The long hours of duty in the wards may reduce our pupils to a condition of servitude, and in many instances it is not for the purpose of giving them more and better training that they are kept on duty long hours, but for the purpose of rendering service and for economising in the working force of the institutions that these are maintained. Working hours should be so arranged as not to exceed nine hours, and should, when possible, be limited to eight hours.

A paper was also read by Miss L. L. Dock on "A National Association for Nurses and its Legal Organisation." This gave a comprehensive plan which included local, State, and national organisations. It is impossible to compress this paper of at least five thousand words into two lines. As it will be printed in full there is no occasion.

Miss McIsaac wrote upon the question "Should Undergraduates be Sent Out to Private Duty?" and answered it with a decided negative, in which she was joined by the other superintendents, who passed a resolution to that effect. Mrs. L. Quintard considered "The Limitation of Pupil Nurses' Duties in Caring for Male Patients," taking the ground that whatever was absolutely necessary to be done was both modest and womanly, but holding that much might be left to orderlies if they were properly trained.

The last paper was by Miss M. E. Smith, on "Uniforms." She deprecated the careless use of this badge of office, and especially the fact that some who have never earned the right to wear it adopt it as a means of securing positions.

The discussions of these papers were pithy and excellent. They will appear in the official report.

By unanimous vote, the fifth article in the constitution was amended in accordance with the proposition of last year.

The following officers were elected for the ensuing year: President, Miss Adelaide Nutting; vice-president, Miss M. E. P. Davis; treasurer, Miss L. L. Drown, Boston, Mass.; secretary, Miss L. L. Dock; auditor, Miss A. S. Brennan; councillors, Miss Marion Smith and Miss McIsaac.

A committee with reference to a National Association of Nurses, the "N.A.N." was appointed as a nucleus of a larger committee on organisation: Miss Dock, Miss McIsaac, Miss Merritt, Miss Brown, and Miss Walker.

The social pleasures of the society consisted of various receptions, visits to hospitals and places of historic interest, and the pleasant luncheons at the Colonnade.

The society adjourned, to meet in Baltimore in February, 1897.

### Talks About Food.

### VII.—BABES AND SUCKLINGS.

There is always something pathetic about the position of a little child, entirely dependent as it is upon others for its sustenance, and with no means of making known its wants, for the first ten or twelve months of its existence, except by a cry which may be taken to mean anything, even—alas! poor baby—temper. But still more pathetic is its lot, when it falls, as it too commonly does, into the hands of people who know nothing of its delicate digestive powers, nor of the needs of the human frame, much less of those of this helpless mite, with its sensitive alimentary canal, and its utter powerlessness to digest what proves to be wholesome food for the robust adult stomach.

It is really wonderful that the poor rear as many children as they do, but when one thinks of the numbers that die (one forgets what the proportion of children lost by an East-end mother bears to the number born, but it is startlingly and terribly large), and of the numbers more that struggle through infancy into a sickly, deformed, stunted childhood, one cannot but feel that there is something sadly wrong about the management of these helpless infants.

Under-feeding, over-feeding, improper feeding—these have slain, and are slaying now, their tens of thousands. It is the easiest thing in the world to kill a baby without resorting to any out-of-the-way means for doing it, as those child murderers, the baby-farmers, know only too well. With them that most useful Society for the Prevention of Cruelty to Children must deal; but, meanwhile, there is much unconscious cruelty to children of which the society can take no note, but which might easily be prevented by other means. The evil is wrought, in fact, by want of thought, and want of knowledge; and it is the business of those who care for these poor little innocent victims, and for the welfare of the race in general, to take care that this want is supplied. We have seldom seen a more useful suggestion than that made by Miss Collet in "Labour and Life of the People," viz., that afternoon classes should be formed for girls of the lower classes who have left school, and are expected to make themselves useful at home—classes in which should be taught cottage cookery, sick nursing, and the management of young children. "I must stay at home to mind the baby," is often the excuse given by girls who have left school and are asked to attend classes. But here, as Miss Collet points out, the baby will be a welcome guest, affording, indeed, a valuable part of the apparatus for practical demonstration lessons. It is quite pleasant to think of poor "baby," whose mother, perhaps, has hurried out to her charing without finding a minute to see to him being carefully washed and dressed, in presence of "Sissie" and her playmates, and laid on a cushion on the floor to kick and crow, while some wholesome, appetising food is being prepared for him.

Even if his mother refuses to lay to heart the lessons taught her by her little girl, we may be pretty sure that the child herself will profit by them when she herself possesses a baby "of her very own." She will learn, for one thing, that saliva is necessary for the digestion of all but the most simple food, and that, therefore, until baby begins to "dribble," it is hopeless to expect him to digest meat, red herrings, or apples. She will learn, too, that milk is highly important, not only for infants, but for all children of tender years, and that it is hopeless to expect her brothers and sisters, or her own children, when she has them, to be anything but stinted, sickly, and weak-toned unless they have some, at least, of the food specially designed for them, and for all young animals, by Providence. She might be told that even at her own age milk is much needed, for it has been found by a doctor, who was also a factory inspector, and who made careful observations and experiments, that between the ages of thirteen and sixteen the factory children grew nearly four times as fast on milk for breakfast and supper as on tea and coffee. She might too be taken to a children's hospital, and there shown the mournful little assemblage of rickety, deformed, and "wasting" children, many of whom might have been perfectly strong and healthy if they had only been properly fed. And she should certainly be told that the habit of handing a child a crust of bread whenever it comes in to ask for it, or if it is a baby of letting it suck for a minute or two whenever it seems restless, is a decidedly hurtful one. Much of this simple teaching might be given in quite an informal way, even without the aid of classes. But where our country population is concerned one may very likely be suddenly pulled up in the midst of a disquisition on the value of milk by a mild "That's very right, ma'am, but it ain't no good a talking, for we can't get milk for love or money." This is, indeed, a poser, and one which we may often meet with in a country village. In some districts there is very little pasture, and in others it pays the farmers better, they say, to send all their milk to London, or even to give it to the pigs, than to sell it in ha'porths to the poor. This is a really melancholy state of things, and those who have at heart the welfare of the poor should do everything in their power to remedy it. A Lady Bountiful who would sell milk cheaply to the poor would do far more good than one who gave away beef at Christmas. Again, cow clubs, such as have been successfully started in certain parts of England, might be commenced—there is room for many more of them; and, as it has been well said, "Clothing and coal clubs are very good, but as a preserver of infantile health a cow diet is worth them all."

True, there are many districts in which the land is almost all arable; but even here goats might be kept. Their milk goes actually further, being richer, than does cow's milk; their keep is by no means a serious item, and there are few cottages where a goat might not easily be kept. We have ourselves made the acquaintance of a goat who was *l'ami de la maison* in the best sense of the word, supplying the children with "milk messes" which were greatly relished, and giving the whole family the unwonted luxury of milk with their tea. If some gentleman living in a country village would begin goat-keeping for himself and gradually supply the cottagers, either by gift or sale, with kids, he might reasonably expect to see a marked difference in the physique of the population before very many years had rolled over his head. Would not this be worth working for? Physical health and strength is the one thing needful if good work is to be done; and who that knows the intimate connection between body and soul can say how much moral evil might not be done away with if the physical frame were made all that it should be—a dwelling-place meet for the sensitive soul within?

### On Certain Aspects of the Nursing Question as Seen in England and Germany.

By a Certificated Midwife.

### GERMANY AND HER SYSTEM.

Wishing to learn at the best possible school, I set out for Vienna, of whose obstetrical teaching I had heard much. But the fates were against me—or, at least, I thought they were then—and I got no further than Stuttgart, where I fell ill directly I arrived, having brought the seeds of my sickness with me. I was inclined to feel disappointed at having my plans overthrown, but in the end I acknowledged that the way in which my feet were forced to tread was the best possible road for reaching my goal. To fall ill in a foreign land and to make my first acquaintance with the inside of her hospitals as a patient was far from agreeable to my wishes, but it produced in me that proper degree of passivity which is always advantageous to a scholar; and so much in German ways was strange, so much distasteful to me, that had I entered on my pupilage at once I should probably have often felt indignant, and sometimes shown myself insubordinate, than which nothing could more have hindered my aptness as a scholar.

As it was, I fell helpless into the arms of a system which nursed me back to life and health on the most scientific principles, at the least possible expense, and with the minimum of inconvenience to either myself or my friends. It also brought me into close contact with some of the first medical men of the place, and when I came out of *Ludwig's Spital* and entered on my four months' course of training at the Government School for Midwives, I carried with me not only the recommendations and advice of the good and clever Dr. Teuffel, but a grateful conviction of the advantages of working on a system, even if it should sometimes run counter to individual feelings. Of my experiences as a "typhoid case" I shall tell later on; though they came first in time, they would, if related now, interrupt the contrast which I wish to draw between the English and German methods of training midwifery pupils.

The very first thing which struck me was the care which the German Government takes of its mothers, as such. As women they often work far too hard, and are treated disdainfully in some ways. But with an eye to population, and an after-glance at future soldiers, the whole country is mapped out into divisions and sub-divisions, and in proportion to its needs so many competent women are appointed to each *gemeinde* or village. In each capital town a school is founded for their instruction, and every district is bound to furnish pupils to be trained in the most useful, necessary, and, it may be, noble profession of midwifery. If there are plenty of volunteers from a district well and good; but if not, the mayor of the little community has to send his own wife. The fees they are asked to pay for this training are but trifling, and board and lodging is given to them. When they get their certificates and go back to their village homes they take with them full instructions as to their duties towards the public and all necessary appliances and medicines. They are under supervision of the medical officer of their district, and have to keep and show an exact register of cases and treatment. Strict rules are also laid down as to how they have to act in abnormal conditions, and what penalties they will incur if anything goes wrong and they have not informed their doctor in due order. They know to whom they must send also; it is not left to chance or choice as with us. Nor does every country doctor profess to be an accoucheur. But the State sees to it that within reach of every midwife there is a qualified *Geburtshelfer*, and to him she not only may, but must, appeal in certain cases.

These schools which, as I have said, are to be seen in all large towns throughout the length and breadth of Germany are what in England we speak of as lying-in hospitals, and the name simply suggests that women go there to be confined. The Germans give them various titles, but always expressing an opposite idea to this. In one town you may hear of the *Hebammen Schule* (midwifery school) in another of the "*Gebär anstalt*" or "institution for births." As a matter of fact, women go to these places to be confined when they are too poor to pay for proper attention at home, just as they do with us. But whereas our primary idea is that the hospital is a charity, and for which the rich must pay in order to help the poor, theirs is that pupils go there to be taught a business, and all expectant mothers are made welcome, and their entrance is entirely free, for they provide the necessary material for practising on. This way of looking at things entirely alters the position of the inmates. If a poor mother goes into a hospital as an object of benevolence she is apt to grumble if she has not all the care and attention she expects. She is loth to give up her individuality and her liberty of action. She clings fondly to her dirty and, perhaps, infected clothes. If she thinks she is badly treated she is free and, indeed, invited to make her complaint heard as against the staff of the hospital to the governors of the hospitals, *i.e.*, the paying public; and perhaps the whole cumbrous machinery of committees special and committees general, visiting ladies, and consulting physicians has to be set in motion to investigate what may be a serious or trivial charge of mal-administration of funds, or neglect on the part of pupils who have paid largely for the privilege of learning there. "The poor mothers are the first consideration," cries the British public, with its fine sentiment; "we put our hands in our pockets that they may be cared for, and away with students, male or female, if they are not subservient to this end." Thus there is a collision of forces and a clashing of interests, which always mean waste of power and a measure of discontent. In Germany there is nothing of this. The State takes on itself the duty of providing—not charitable houses where the poor may lie-in—but a regular succession of midwives competent to attend mothers of all ranks in their own homes. It accordingly builds and furnishes a school-house on the best-known plan, seeks out the best qualified medical man as teacher, and places him at the head of the institution; and, paying him well according to German ideas, lays on his shoulders the entire responsibility of—1st, the competency of the midwives sent out from the school; 2nd, the life or death of the mothers who are confined there; 3rd, the due economy of its conduct. The results and statistics are duly tabulated and published, and there is a periodical inspection by a Government official. Then, having done its part, it needs but one thing—human material to work on, and for this it appeals to the public. "Come when you will," says the State in effect, "we will provide you with food and clothes during your months of expectation, and skilled care for your hours of danger. We ask in return that you will submit to be used as text-books for our pupils' teaching. For our own credit's sake, as well as for the common weal, we take the best care of your health and safety that our experts in the science of childbirth know how to give."

It may be distasteful to us English to think that, because women are poor or unfortunate, they may be used as so much material on which pupils may practise. To me this system brought many qualms. But I remembered the alternative—women arriving at our London hospital doors already in the pangs of travail, and babies that I had seen born in a cab, or perhaps on the threshold of the house while the cabman looked on. I remembered, too, the high temperatures, the frequent infection brought in from filthy homes, the hurries and worries endured by young girls in their shame and distress, going round from one governor's house to another begging for an order of admission, and then telling untruths to get inside the hospital for one night only, to be sent out the next day when it was found there might be a week or more to wait before their time was come. And, balancing one evil against another, I swallowed down my sentiment and, looking facts straight in the face, I acknowledged that the German woman had the best of it.

### A Book and its Story.

### "THE RED BADGE OF COURAGE."*

This is the story of an episode in the American Civil War; it is not a novel in the particular sense of the word, as that word is used in 1896. There is no love nor love-making here in all the two hundred pages of Mr. Crane's book. There is neither discussion of sex question nor of equivocal morals. But if it lacks all the modern essentials of a novel, "The Red Badge of Courage" holds its own as one of the most forcibly-written romances of the day. In its own way it is as powerfully-drawn a war picture as is Zola's "Débâcle"—the scenes depicted are not less vividly brought before us. In "The Red Badge of Courage" we are brought face to face with the big realities and the trivialities of warfare, with the sordid details of a soldier's life in its heroic and in its unheroic aspects. Mr. Crane lays bare to us the inner workings of a young warrior's mind, as they have not been shown before, and "The Youth," as he designates his hero, is a flesh and blood reality to his readers, an actual person whom we feel we know; whom we pity, condemn, and criticise in a thousand ways, because of this very actuality. We are introduced to him in this way: "The Youth was in a little trance of astonishment. So they were at last going to fight. On the morrow, perhaps, there would be a battle, and he would be in it. For a time he was obliged to labour to make himself believe. He could not accept with assurance an omen that he was about to mingle in one of those great affairs of the Earth. He had, of course, dreamed of battles all his life—of vague and bloody conflicts that had thrilled him with their sweep and fire. In visions he had seen himself in many struggles. He had imagined peoples secure in the shadow of his eagle-eyed prowess. From his home his youthful eyes had looked upon the war in his own country with distrust. It must be a sort of play affair. Tales of great movements shook the land. He had read of marches, sieges, conflicts, and he had longed to see it all. He had burned several times to enlist, but his mother had discouraged him." She had affected to look with some contempt on the quality of his war ardour and patriotism. Nevertheless, he had gone to a town that was near his mother's farm, and had enlisted in a company that was forming there. When the youth returned home after this heroic act, he found his mother milking the brindle cow. "Ma, I've enlisted," he said to her diffidently. There was a short silence. "The Lord's will be done, Henry," she replied faintly, and had then continued to milk the brindle cow.

On the eve of his starting, the admonitions of the mother to the warlike son, clothed in blue and gold, were of a complex order; an exhortation, commanding him to stand by the colours he had chosen, wound up with the words, "Don't forget about the socks and shirts, child; and I've put a cup of blackberry jam with yer bundle, because I know yer like it above all th'ng'. Good-bye, Henry. Watch out, and be a good boy." The speaker's upraised face was stained with tears; her spare form w is quivering. The son bowed his head, and went on, feeling suddenly ashamed of his purpose.

Monotonous life in a camp for many months followed our hero's enlisting. The "real war" of death struggles and privation of which he had dreamed was, during that time, far off from realisation. Since his regiment had come to the field the army had done little but "sit still and keep warm."

News of definite fighting brought definite feelings to the youth's mind. "Think any of the boys 'll run?" he asked a soldier who had seen action. That question revealed the questioner's mind. He was a hero, a soldier, in theory only. The eve of the grand meeting of the foes is uniquely pictured: "The sun spread disclosing rays, and, one by one, regiments burst into view like armed men just born of the earth. The youth perceived that the time had come. He was about to be measured. For a moment he felt in the face of his great trial like a babe, and the flesh over his heart seemed very thin. . . . But he instantly saw it would be impossible for him to escape from the regiment. It enclosed him. And there were iron laws of tradition and law on four sides. He was in a moving box. . . . He became not a man, but a member. He felt that something of which he was a part—a regiment, an army, a cause, or a country—was in a crisis. He was welded into a common personality which was dominated by a single desire. For some moments he could not flee, no more than a little finger can commit a revolution from a hand." But the thought of flight was only deterred by circumstances; it was the dominating intention in the mind of the youth. We follow him as he passes in under fire. Mr. Crane holds one in breathless excitement, as he depicts The Youth leading his company into the region of shells, which hurtled over his head with long wild screams. The word painting is graphic—inimitable. "As he listened he imagined these shells to have rows of cruel teeth that grinned at him." They were too much for him. Eventually the youth flew the scene of terror. The romance of the tale centres round this flight; it is the episode *par excellence* of the narrative, and the hero's vindication of his conduct to his accusing conscience is a marvellous bit of realism. A certain moth-like quality within him kept him in the vicinity of the battle. He had a great desire to see and get news. He wished to know who was winning. "He told himself that, despite his unprecedented suffering, he had never lost his greed for victory. A defeat for the army, he said, in a half-apologetic manner to his conscience, might mean many favourable things for him. The blows of the enemy would splinter regiments into fragments. Many men of courage would be obliged to desert the colours and scurry like chickens. He would appear as one of them. They would be sullen, brothers in distress, and he could then easily believe he had not run any further or faster than they." The tragedy of a gory battlefield palls before the mental tragedy of cowardice and desertion The stabs of the youth's conscience were a greater thing than the sword-thrust which he avoided. Left to himself in a distant wood, he pacifies the best he may the instincts of his better self which torment him. He has escaped one battle to confront one of greater magnitude. He curses himself and his conduct. Afterwards, the skirmish over, on rejoining his regiment, he is tormented with fears lest the wounded men should see the letters of guilt he felt burned upon his brow. "At times he regarded the wounded in an envious way. He conceived persons with torn bodies to be peculiarly happy. He wished that he, too, had a wound—a red badge of courage." As the narrative proceeds, we see The Youth in another light. He faces battle, tearing the standard from the dead bearer's hands. He is a soldier, no longer one of "traditional" courage. His flight is known to himself alone; out of it he evolves a different future.

The story of the soldier has something in it beyond mere word painting and literary merit; it is a history with a suggestion which we can each take to ourselves. The author concludes in these words: "He (The Youth) found that he could look back upon the brass and bombast of his earlier gospels, and see them truly He was gleeful when he discovered that he despised them. With this conviction came a store of assurance. He felt a quiet manhood, non-assertive, but of sturdy and strong blood. He knew that he would no more quail before his guides, wherever they should point. He had been to touch the Great Death, and found that, after all, it was but the Great Death. He was a man."

* "The Red Badge of Courage." Stephen Crane. The Pioneer Series. (London: Heinemann. 1896.)

### Everybody's Opinion.

[Correspondence on all subjects is invited, but we cannot in any way be responsible for the opinions expressed by our correspondents. No communications can be entertained if the name and address of the correspondent is not given, or unless one side of the paper only be written on.]

### NURSES AND LAY COMMITTEES.

"A Superintendent of District Nurses" writes: I am glad that "Criterion" acknowledges the right of the poor to have a nurse if one is to be had, and that she exists solely for their benefit. But I would ask if this is not a part of the "management" of which he complains? As to the district nurse "pressing her unsolicitated services on poor people," I should be sorry to believe that any nurse would do so, unless, in a case where she felt sure the patient needed her help, and only declined it because afraid of a nurse. Such cases are by no means uncommon, and all honour to the nurse who has patience and tact to help such a person in spite of apparent want of appreciation. But if some enthusiasts have not sufficient self-respect to prevent their "canvassing," I would suggest they might well be left to work out their own punishment—in all probability it is just because they are not nurses that they do it—it is certainly not necessary or desirable that they should. As to "Criterion's" next complaint, it is equally unnecessary for a district nurse to make any report to her committee that might lessen confidence between a medical man and his patients; but, as she must give some account of her work to those who employ her, would it not be possible for the doctor (in the village) to try and show the committee the objections to any plan that he felt worked badly? If the reports of a nurse's work were of a general character—that is, more importance being attached to the amount of time she spent in work than to what she did—many difficulties would be avoided.

### HELPERS OR RIVALS?

"Matron" writes: Your paper dealing with the subject of nurses and doctors concerns every member of the profession very closely. As your article infers, women engaged in private nursing are the chief offenders. To trained nurses, and anyone else who understands the matter, such conduct as you describe points very clearly to inadequate training, or none at all. Of course, much of the disrepute is due to individual unsuitability for the work; but it is within the power of heads of institutions, doctors, and the public to insist that these women have the chance of becoming what the latter pay liberally for, and expect to get, viz., properly trained nurses. But what is a trained nurse?

"M. H." writes: In reference to your article in The Hospital of February 22nd, entitled "Helpers or Rivals?" I feel bound to say something in defence of my sister nurses. I have had to do with the training of nurses, and have seen many start their career as private nurses after their full term of hospital training, but I can honestly say, with very few exceptions indeed, they have all stepped thoughtfully and carefully into their new sphere, feeling, as I have so often had occasion to tell them, too little self-confidence instead of too much. As for their "contempt for the general practitioner," that is really absurd, and any general practitioner who will take the trouble to treat the disease of his patient as more important than the idiosyncrasies of the family connections, and will give the nurse proper instructions as to the treatment he requires, will find those instructions carried out in the majority of cases to the minutest detail, and the nurse ready with a concise and clear report for him at his next visit. The general practitioners which nurses find it so really hard to work for are those who come into the house in an apologetic kind of way, rubbing their hands and smiling affably at all the inmates in turn, who listen to the account of the patient's progress from the patient himself, as well as all his sisters, cousins, and aunts, who acquiesce in everything they each in turn recommend, quite oblivious to the fact that a trained nurse has sat up with the patient all night, and therefore ought to know a little more of the progress of symptoms than these interfering busybodies. These are the men who are liable to make a nurse look with contempt on the general practitioner, and regard him as merely a collector of fees, who, even if he has the knowledge has not the courage to treat his patient scientifically as a case, but rather as simply a means of augmenting his own livelihood. My experience of nurses is this—The more real knowledge they have the more convinced are they of their own ignorance, and the more timid do they become of encroaching upon the doctor's preserves. If there is an evil, as the writer of the article in question really seems to feel there is, I can suggest but one remedy, *i.e.*, be careful to have a nurse with a thorough training at her back (three years at least in hospital), choose her from the more highly educated classes, and give her a chance of nursing her patient as she has been taught to do. Be loyal to her. Give her your orders, and expect to find them carried out. No good nurse will ever grumble at the minuteness of the doctor's orders, or his care in having them executed correctly, unless she happens to be a half-trained, uneducated woman; in fact, a woman to whom "a little knowledge is indeed a dangerous thing."

### NURSING AT SMALLER PROVINCIAL HOSPITALS.

"A Matron" writes: Having been for the past twelve years the superintendent of a small provincial hospital with an average of about twenty-five patients, I shall be glad to give "An Honorary Secretary," who writes on the subject of "Nursing in Smaller Provincial Hospitals" in your issue of February 15th, the benefit of my experience. I have tried different arrangements with regard to the nursing, and have at last arrived at a satisfactory solution of the problem, "What is to be done in order to supply what we so much need, viz., trustworthy nurses who are willing to act in a subordinate position?" My answer is—Train your own nurses. Out of the numerous applicants for vacancies for probationers choose those who either in their own homes or elsewhere have had some experience of practical work. After a month's trial, if suitable, bind them to serve for three years, with a progressive salary (£10, £14, £18). I find that a third, or even second year probationer, who has been trained from the first to use her discretion and tact in dealing with the patients and their visitors, or in any sudden emergency, makes a much more dependable nurse than one who has worked for three years in a subordinate position in a large hospital where either a "sister" or a resident is always at hand. My probationers always give me voluntary and willing help in the dispensing for in and out-patients, care of the linen, &c., so that their work, though more varied, is as constant as that of a probationer in a large hospital. If "An Honorary Secretary's" suggestion were carried out, in my opinion the managers of the large hospitals would be indebted to the smaller hospitals for the board and lodging of a succession of tried nurses who would be more difficult to teach than beginners because less receptive. Ten years ago I was under the impression that a nurse could be thoroughly trained in a large hospital only, but experience has proved to the contrary. Trained nurses are so much needed in these days for private and district nursing, as well as for work in poor-houses, that it seems a pity to waste the valuable teaching which can be given in a small hospital by employing only trained or half-trained nurses.

### Death in Our Ranks.

We much regret to announce the death, on February 23rd, from pneumonia, of Nurse Rimmer, at the Infirmary, Lancaster, after only a week's illness. Nurse Rimmer received her training at the Leeds General Infirmary, and had been for fourteen years staff nurse at Lancaster, where she had won for herself the sincere esteem and respect of patients and fellow-workers, and where her loss will be much felt. At the funeral, the expenses of which were borne by the committee of the infirmary, there were many beautiful flowers, including a cross of tulips and blue and white hyacinths from the hon. medical staff.

### Notes and Queries.

### Queries.

(167) *Monthly Nurse.*—Please tell me if I can legally claim my fee under the following circumstances. I am a ladies' monthly nurse (L.O.S. diploma), and a case for which I was engaged came off a month before the time. I was not sent for, although I could have gone, and no notice was taken of a letter in which I offered to take half the fee. It is a great loss to me, and I shall be much obliged by your advice.—*Nurse Annie.*

(168) *Private Asylums.*—Can you give me the addresses of small private homes where male patients can be received for a moderate sum?—*Nurse.*

(169) *Training.*—I want to enter a general hospital in London for two or three years' training. Where shall I apply? My age is 22.—*M.R.*

(170) *Training.*—Can you give me the names and addresses of hospitals to which I can apply for admission as probationer? My age is 22.—*K. C.*

(171) *Asylum Attendant.*—Can you give me any information about pensions for asylum attendants? I have always understood that county asylums granted pensions after long service, but I am told that this is not so under the County Council.—*Pension.*

(172) *Home of Rest for Nurses.*—Can you give me the address of the Home of Rest for Nurses at Hastings, and tell me the terms for two nurses going together?—*Nurse R.*

(173) *Maternity Nurse.*—Will you give me the names and addresses of hospitals where a maternity nurse aged 35 could get general training, and also do you know of any Catholic institution which takes maternity nurses on its staff?—*Victoria.*

(174) *Lodgings.*—Can you tell me of any rooms not far from the London Hospital where I could stay for a day or two? I know no one in London and am coming up to see the matron. Would you also tell me the cab fare from King's Cross to the hospital?—*Country Girl.*

(175) *Male Nurse.*—Will you please tell me how I can obtain training as a nurse? I have attended ambulance lectures, and should like to take up nursing as a means of livelihood.—*Elgin.*

(176) *Training.*—Can you tell me of any hospital or district nursing association which would take me as probationer? I am 39, but very strong and active.—*Widow.*

(177) *New York.*—Can you kindly tell me how to set about gaining admission into one of the New York hospitals as nurse? I should also be glad to know something of the hours on and off duty in American hospitals.—*Emigrant.*

(178) *Epileptics.*—Can you tell me of any home between Cornwall and Exeter where an epileptic patient could be received at very small cost?—*Sister D.*

(179) *District Nurses.*—Will you kindly tell me whether there are district or parish nurses, not from an institute, at Plymouth, Brighton, Boston (Lincs.), Nottingham, Worcester, &c., &c., and the name and address of the secretary?—*Constant Reader.*

(180) *Monthly Nurse.*—I am desirous to enter a lying-in hospital to train as a monthly nurse. Will you tell me if in such a hospital the nurse is taught how to act in an emergency, and what text-books would you recommend to a student of midwifery and a monthly nurse?—*Searcher.*

### Answers.

(167) *Monthly Nurse* (*Nurse Annie*).—As you were engaged to attend the case in question, and were ready to carry out the engagement on your part, you will be entitled to damages for not having been employed pursuant to the contract, the measure of damages being the amount of your fee.

(168) *Private Asylums* (*Nurse*).—You will find a list of "private asylums and licensed houses" on p. 485 in "Burdett's Hospital and Charities Annual" (Scientific Press, 428, Strand, W.C.). Apply for particulars of terms of admission to the superintendents.

(169) *Training* (*M. R.*).—Such questions as yours are being constantly answered in these columns. The London general hospitals do not take probationers under 25 years of age as a rule. You might apply at some of the children's hospitals, where younger candidates are accepted. At the Poplar Hospital for Accidents probationers are now received at 22. Get "How to Become a Nurse" (Scientific Press, 428, Strand, W.C.), in which you will find all the information you need.

(170) *Training* (*K. C.*).—See reply above under this head, which answers your questions.

(171) *Asylum Attendant* (*Pension*).—The Royal National Pension Fund for Nurses and Hospital Officials will meet your needs. Write for particulars to the Secretary, Royal National Pension Fund for Nurses, 28, Finsbury Pavement, London, E.C.

(172) *Home of Rest for Nurses* (*Nurse R.*).—We do not know of any special home for nurses at Hastings. There are several convalescent homes both at Hastings and at St. Leonards. Do you mean one of these?

(173) *Maternity Nurse* (*Victoria*).—You will find full lists of London and provincial general hospitals in Burdett's "Hospital and Charities Annual," Scientific Press, 428, Strand. Make a selection and write to the matrons of the various institutions for particulars. Do you mean Roman Catholic? In England very few public institutions make any sectarian distinctions in filling vacancies on their staffs.

(174) *Lodgings* (*Country Girl*).—We do not know of any suitable place to recommend very near the London Hospital. We believe nurses find comfortable quarters at the Nurses' Hostel, 27, Percy Street, Tottenham Court Road, W.C. You might also try Miss Ely, 25, Norfolk Terrace, Hyde Park, W.; or Miss Culverhouse, 18, Royal Avenue, Chelsea, S.W. The fare from King's Cross to the London Hospital is 2s. You will find a list of cab fares in the A B C Railway Guide.

(175) *Male Nurse* (*Elgin*).—There is no school for training male nurses in England as yet. You might write to the Hamilton Association for Providing Trained Male Nurses, 57, Park Street, Grosvenor Square, W.

(176) *Training* (*Widow*).—Your age will be an obstacle unless you can afford to pay for your training. Have you applied to provincial hospitals? Watch the advertisements in our columns, or advertise there yourself.

(177) *New York* (*Emigrant*).—Write direct to the matrons of the various hospitals in New York, of which you will find a full list in Burdett's "Hospital Annual" (Scientific Press, 428, Strand, W.C.). "The Hospital Nursing Supplement" for September 21st, 1895, contains an account of the New York City Training School for Nurses, with particulars about hours, &c. You can get back numbers from the publishers, 428, Strand, W.C.

(178) *Epileptics* (*Sister D.*).—We do not know of any special home in the neighbourhood of Exeter. You will find a list of homes and charities in Burdett's "Hospital Annual." Why not write to the Society for Employment of Epileptics, 12, Buckingham Street, Strand, W.C., or to the Meath Home of Comfort, Miss Annie Cazenove, Ravenleigh, Betchworth, Surrey?

(179) *District Nurses* (*Constant Reader*).—Your query is not at all clear. What do you mean by "not from an institute"? Most large towns in these days possess district nurses. The Q.V.J.I. has a branch at Worcester. You can find the addresses of District Nursing Societies and Institutes in Burdett's "Hospital Annual."

(180) *Monthly Nurse* (*Searcher*).—Get "How to Become a Nurse" (Scientific Press, 428, Strand, W.C.) and read the chapter on "Midwifery and Monthly Nursing"; then write to the various hospitals for particulars of terms and training. In the same book you will find a list of text books likely to be useful to nurses.

### Where to Go.

The Dowdeswell Galleries, 160, New Bond Street, W.
Brittany and the Norfolk Broads. By W. Y. Laidley.

Mr. Laidley's charming collection of paintings on view during March at the above gallery is very well worth a visit. There are only 100 pictures in all, but they show the artist in various ways as a landscape, figure, and subject painter. As a figure and a subject painter Mr. Laidley does not appear at great advantage; in the former field he has no individuality of treatment, and in the latter the same want is felt. As a landscape painter he exhibits great talent and originality. His canvases grow on one as one becomes more familiar with them. In technique and in scheme alike they are admirable. Their luminosity and sense of atmospheric effect stamp them one and all as being drawn in the open. They are imaginatively and suggestively painted, and yet in no one instance among the 100 here exhibited is there any disregard on the artist's part of the strictest sense of value. Especially is this noticeable in the Brittany pictures, among which three or four stand out as examples of exceptionally fine landscape work.

Trained Nurses' Club, 12, Buckingham Street, Strand.—Complimentary benefit. A musical and dramatic entertainment will be given (by kind permission of the committee) on Wednesday and Thursday evenings, April 15th and 16th, at half-past seven, by Mrs. Nichol, Secretary of the Midwives' Institute and Trained Nurses' Club. Tickets, 2s. 6d. each, can be obtained at the club from the Hon. Treasurer, Miss Brierly.

North London Nursing Association, 413, Holloway Road, N.—The annual meeting of this association is announced to take place on Monday next, March 9th, at eight p.m., when an address will be given by Mrs. Dacre Craven. This home for district nursing was first opened in 1877, and was the first branch home of the Metropolitan and National Nursing Association. The work has been supported entirely by local effort, but additional funds are now urgently needed.

Hospital for Sick Children, Great Ormond Street.—Special Lenten addresses are being given in the beautiful chapel of this hospital on Wednesday afternoons at half-past four. On March 11th the Rev. W. F. H. Randolph, vicar of St. Andrew's, Croydon, preaches on "The Worker's Strength"; on March 18th the address will be by the Rev. the Hon. John Stafford Northcote, vicar of St. Andrew's, Westminster, on "Pain Teaching Love"; and that on March 25th will be by the Rev. Herbert Jeaffreson, of St. Augustine's, Kilburn, on "Reverence Towards Children."

Master Gladstone Bell's Violoncello Recital in aid of the funds of the Surgical Aid Society took place at the Queen's Hall, Langham Place, W., on Saturday afternoon last. The concert was under the patronage of the Right Hon. the Lord Mayor and the Lady Mayoress, and the Hon. Sir Francis and Lady Jeune. Master Bell was assisted by the Schartau Part Singers. The boy violoncellist played in all ten pieces. He exhibited a talent of no mean order, and delighted his audience with the tone and force of his playing. He has, besides, a good memory, and plays without his notes. As far as the music of the concert went, it was admitted to be a success, but considering the claims of the excellent society in whose favour the recital was held, one would have been glad to see a more active exhibition of sympathy from the public in lieu of many vacant seats.

